# Demographic and clinical factors correlated with clinical outcomes among people with HIV treated by antiretroviral therapy: a retrospective cohort study

**DOI:** 10.1186/s12879-024-09406-w

**Published:** 2024-05-22

**Authors:** Yuwei Li, Hengli Liu, Shuangmei Zhang, Yanyun Zhang, Haiyang Wang, Huanhuan Zhang, Xia Li

**Affiliations:** 1 Beijing Chest Hospital, Capital Medical University, Fengtai District, Xitoutiao Road No. 10, Beijing, 100069 China; 2 Beijing Tuberculosis and Thoracic Tumor Research Institute, Tongzhou District, Machang Road No. 97, Beijing, 101199 China; 3 Department of Infectious Diseases, Yunnan AIDS Care Center, Yunnan Infectious Disease Hospital, Shi’an Road 28 Kilo, Taiping Town, Kunming City, Anning, 650108 Yunnan Province China; 4 School of Public Health, Dali University, Wanhua Road No. 22, Xiaguan Town, Dali City, Dali Prefecture , 671003 Yunnan Province China

**Keywords:** HIV, AIDS, Peak CD4, Antiretroviral therapy, Hazard ratio

## Abstract

**Background:**

As is known, CD4 cell count is a significant parameter predicting HIV progression, opportunistic infections and death in HIV-infected individuals, as well was an important indicator for initiating antiretroviral therapy (ART). In China’s National Free Antiretroviral Treatment Program, people with HIV (PWH) on ART can receive a CD4 count test at least once every six months. Importantly, the baseline CD4 count (before ART initiation) is significantly correlated with ART and even prognosis, but the influence of the peak CD4 cell count on ART and/or clinical outcomes is still unknown.

**Methods:**

A retrospective study was conducted among 7965 PWH who received ART from October 2003 to September 2022 at Yunnan Infectious Disease Hospital. Clinical features and laboratory data were collected and analyzed by Chi-square test, univariate and multivariate Cox regression analysis. After elimination of confounding variables, multivariate Cox regression analysis was performed to identify survival-related factors.

**Results:**

Of a total of 7965 PWH in the ART treatment cohort who met the inclusion and exclusion criteria, 7939 were finally included in the subsequent analyses. First, it was found that the proportion of clinical variables, including sex, age distribution, interval from diagnosis to ART initiation, marital status, and others, was significantly different between the living and dead groups (*P* < 0.05). Impressively, significantly more PWH had the higher level of baseline, peak and recent CD4 cell counts in the living group compared to those in the dead group. Due to multicollinearity effect, after excluding confounders, the following factors were found to be significantly associated with mortality by multivariate Cox regression analysis: (1) male sex (hazard ratio (HR) = 1.268 [1.032, 1.559]; *P* = 0.024); (2) time from HIV confirmation to ART initiation ≥ 6 months (HR = 1.962 [1.631, 2.360]; *P* < 0.001); (3) peak CD4 cell count: Peak CD4 < 100cells/µL group (HR = 16.093 [12.041, 21.508]; *P* < 0.001), 100cells/µL ≤ x < 200cells/µL group (HR = 7.904 [6.148, 10.160]; *P* < 0.001), 200cells/µL ≤ x < 350cells/µL group (HR = 3.166 [2.519, 3.980]; *P* < 0.001), 350cells/µL ≤ x < 500cells/µL group (HR = 1.668 [1.291, 2.155]; *P* < 0.001).

**Conclusion:**

Interestingly, patients in male, time from HIV confirmation to ART initiation ≥ 6 months, or peak CD4 count < 500 cells/µl had inferior clinical outcomes, in other word, a lower peak CD4 cell count significantly increased the risk of death, and peak CD4 cell was independent in predicting the overall survival of PWH. It is important to promote “early diagnosis and treatment of HIV” and regularly monitor CD4 levels in HIV/AIDS to evaluate the efficacy of ART and immune reconstitution, and optimize the ART regimen in time to further reduce the mortality of PWH.

## Introduction

As antiretroviral therapy (ART) continues to be promoted and optimized, mortality associated with HIV/AIDS has decreased. In China, although the number of HIV-related deaths increased from 5,485 in 2007 to 18,737 in 2019, the overall morality rate decrease from 10.9 to 4.3% [1].As a chronic disease, HIV/AIDS is widely recognized as manageable worldwide [2]. In some cases, life expectancy for with HIV (PWH) on ART is estimated to be close to that of the general population, but ART failure and death are still relatively common among PWH who have low CD4 cell count at baseline (pre-ART).

When PWH have low CD4 cell counts, AIDS, opportunistic infections, or death are more likely to occur. It provides essential evidence to assess the efficacy of ART and immune reconstitution and was once an important indicator for initiating ART [3]. Experts from different eras have different recommendations on when to start ART in PWH. ART eligibility according to Chinese Guidelines [4–6] has expanded from initiation at CD4 < 200 cells/µl in 2005 to a universal “treat-all” approach in 2021. Overall, there is an increasing tendency to actively initiate ART to protect patients from severe immune failure. Meanwhile, the Department of Health and Human Services (DHHS) and the European AIDS Clinical Society (EACS) guidelines also recommend that clinicians should initiate ART immediately, regardless of the baseline CD4 cell count [7, 8]. Although recent versions of guidelines in a number of countries recommend immediate initiation of ART for HIV/AIDS, the previous recommendations have had a noticeable impact on the timing of ART initiation and CD4 cell counts in PWH over the past two decades. The PWH included in this study initiated ART at different stages and their CD4 cell counts varied widely, which is of good research value.

Data from studies suggest that PWH with a pre-ART baseline CD4 of > 500 cells/µl may have a lower risk of death [9]. Meanwhile, studies have shown that there was a higher mortality rate among patients with lower baseline CD4 counts [10]. Several studies have also confirmed the important role of CD4 cells in helping PWH to defend against tuberculosis [11, 12], or in measuring the size of their HIV reservoirs and accessing the degree of immune failure, immune activation status, prognosis, and risk of non-AIDS-related inflammation [13–15]. However, it is unclear whether and to what extent there is also an effect of CD4 peak during the ART process on the survival status of PWH.

We used data from the Chinese National HIV Drug Resistance Surveillance and Monitoring Network since 2003 and the Yunnan Provincial Antiviral Therapy Assisted Management System since the establishment of the database in 2003. In addition, demographic characteristics and clinical characteristics are examined in relation to AIDS deaths, we focused on whether and to what extent the peak value of CD4 cell count affects mortality in PWH.

## Materials and methods

### Study population and data collection

This study is a retrospective cohort study of all 7965 PWH on ART from 2003 to 2022 at Yunnan Infectious Disease Hospital (the largest ART clinic in Yunnan Province). All PWH were diagnosed by enzyme-linked immunosorbent assay and western blot test. Viral load is a key indicator to assess the effectiveness of ART treatment. HIV-RNA testing is performed before ART treatment and every 3 months after ART treatment. Six-monthly follow-ups were provided to all patients, all participants were ARV-naïve at the time of their inclusion in the study, observations were stopped for patients who could not be followed up or discontinued ART. Patients who were lost, died, or no longer received ART during the 6-month follow-up period were excluded from the data.

Demographic information (sex, age, marital status, date of HIV diagnosis and ART initiation, survival status, and date of death), clinical characteristics, and peak CD4 cell counts were collected. Patients were classified into four groups (< 100, 100–199, 200–349, and 350–499 cells/µl) based on initial CD4 cell counts. 7965 patients were included, and data from 26 patients were excluded cause of logical errors. Data from 7939 patients were analyzed, including 7365 patients who survived and continued ART and 574 patients who died.

### Definition of outcomes

Peak CD4 is defined as the highest CD4 cell count of the patient’s previous CD4 test results per PWH from the start of ART until the end of data collection in September 2022 (the cut-off for peak CD4 count for deceased patients was the date of death). Baseline CD4 cell count is a patient’s CD4 cell count prior to ART initiation. The most recent CD4 cell count is defined as the most recent CD4 cell count test result of the PWH through September 2022 (for deceased patients, the cut-off date is the date of death), hereafter referred to as the most recent CD4.

### Statistical analysis

Descriptive statistics were used to analyze and compare categorical variables, and chi-squared tests were used to describe cohort characteristics. Univariate and multivariate Cox regression models were used to assess the association between covariates and mortality risk, with missing and logical error data removed. Variables associated with mortality included: sex (male or female), age, ART duration (</≥ 6 months), marital status (married or partnered, divorced, widowed, single, unknown), mode of transmission (mother-to-child transmission, injection drug users (IDUs), blood transfusion or blood product transmission, homosexual contact, heterosexual contact, unknown), CD4 cell peak count groups (< 100, 100–199, 200–349, 350–500 cells/µl). A p-value of less than 0.05 is considered statistically significant. All statistical analyses were performed using SPSS Statistics 20.

## Results

### Baseline characteristics

**Fig. 1:**
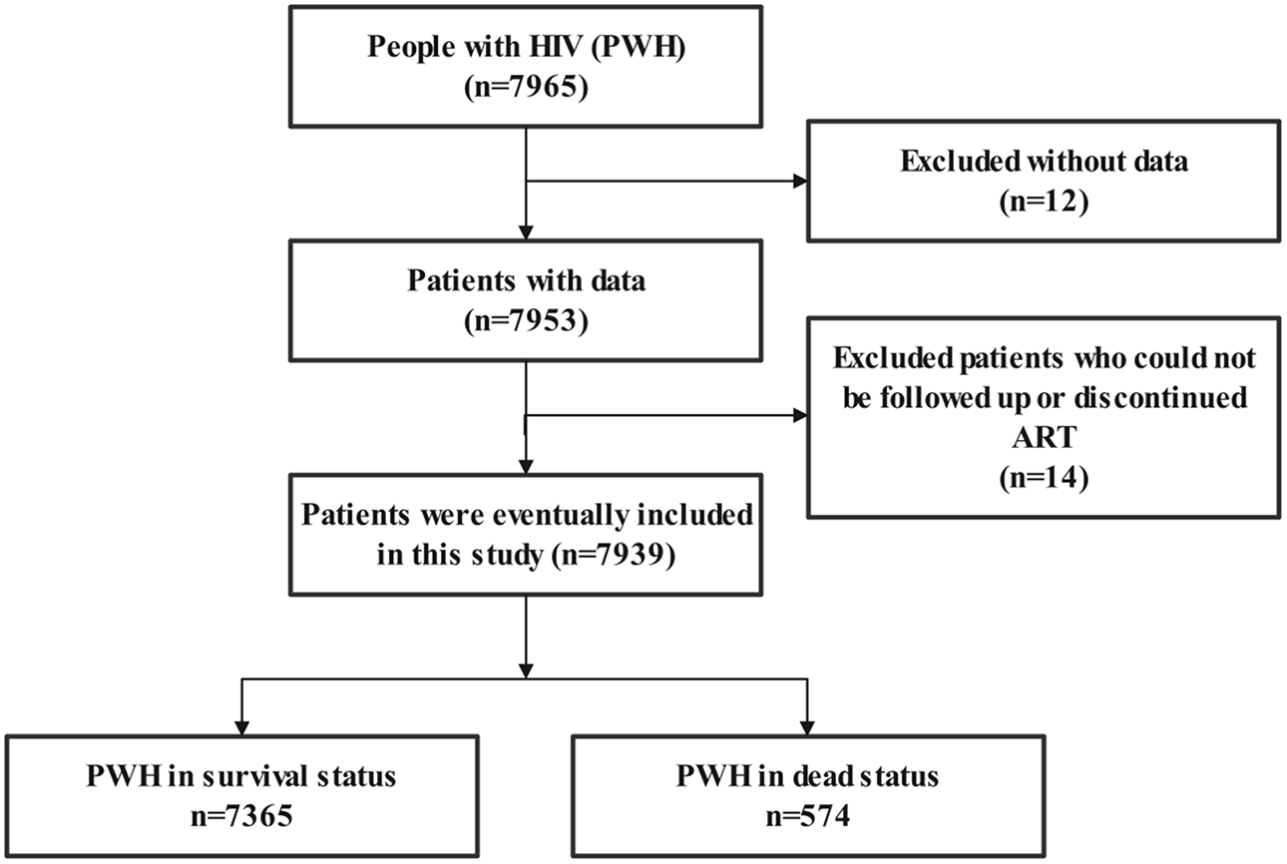
Study flow diagram

A total of 7965 PWH were included for ART from October 26, 2003 to September 7, 2022. As shown in Figs. 1 and 7939 patients were included in the multivariate Cox regression analysis, 574 (7.2%) died, 7365 (92.8%) survived and remained on ART, and 495 (6.2%) had virologic failure (HIV-RNA > 1000 copies/mL).

In this study, 5433 (68%) PWH were male. The median age was 44.5 years (IQR 35.7–52.6) with a range of 2.7 to 87.5 years. The mean duration of ART was 7.4 ± 4.24 years. 3169 (40%) subjects were 30–45 years old and 2910 (37%) were 45–60 years old. There were 5909 (74%) patients who started ART within six months after HIV diagnosis and 5385 (68%) patients who remained on ART for more than five years after initiation. Regarding marital status, 4015 (51%) participants were married or had regular sexual partners, and 2877 (36%) were single. Regarding the mode of transmission, 4043 (51%) cases were heterosexual contact, 1685 (21%) were homosexual contact, and 1036 (13%) were IDUs. Statistical analysis was performed by chi-squared test, and all P values were less than 0.05 (Table 1).

**Table 1:** demographic characteristics and clinical features of 7939 HIV/AIDS

	Total *n* = 7939	Alive *n* = 7365	Dead *n* = 574	*P*-value
Gender				< 0.010
Male	5433 (68.43%)	4983 (62.77%)	450 (5.67%)	
Female	2506 (31.57%)	2382 (30.00%)	124 (1.56%)	
Age distribution				< 0.010
Median age (Q1, Q3)	44.5 (35.7, 52.6)	44.2 (35.2, 52.1)	48.9 (41.675, 59.675)	
< 30yrs	954 (12.02%)	938 (11.82%)	16 (0.20%)	
30-44yrs	3169 (39.92%)	2974 (37.46%)	195 (2.46%)	
45-59yrs	2910 (36.65%)	2688 (33.86%)	222 (2.80%)	
≥ 60yrs	932 (11.74%)	792 (9.98%)	140 (1.76%)	
Diagnosis to ART initiate interval				0.007
< 6 months	5909 (74.43%)	5509 (69.39%)	400 (5.04%)	
≥ 6 months	2030 (25.57%)	1856 (23.38%)	174 (2.19%)	
Marital Status				< 0.010
Married /regular sexual partners	4015 (50.57%)	3641 (45.86%)	374 (4.71%)	
Divorced/ widowed	818 (10.30%)	749 (9.43%)	69 (0.87%)	
Unmarried	2876 (36.23%)	2755 (34.70%)	121 (1.52%)	
Not available	230 (2.90%)	220 (2.77%)	10 (0.13%)	
Route of transmission				< 0.010
Mother-to-child transmission	144 (1.81%)	143 (1.80%)	1 (0.01%)	
IDUs	1070 (13.48%)	861 (10.85%)	209 (2.63%)	
Men Who Have Sex with Men	1685 (21.22%)	1660 (20.91%)	25 (0.31%)	
Heterosexual transmission	4043 (50.93%)	3801 (47.88%)	242 (3.05%)	
Not available	997 (12.56%)	900 (11.34%)	97 (1.22%)	

### Analysis of laboratory data

Chi-squared analysis was performed on the laboratory data of 7939 patients included in the study (baseline CD4: X2 = 164.705, *P* < 0.01; peak CD4: X2 = 1092.856, *P* < 0.01; most recent CD4: X2 = 1287.668, *P* < 0.01, Table 2), showing a significant difference in survival based on CD4 cell counts at different time points.

**Table 2:** CD4 cell counts at different times

	Total *n* = 7939	Alive *n* = 7365	Death *n* = 574	*P*-value
Baseline CD4				< 0.010
< 100/µL	1807 (22.76%)	1589 (20.02%)	218 (2.75%)	
100/µL-199/µL	1498 (18.87%)	1345 (16.94%)	153 (1.93%)	
200/µL-349/µL	2400 (30.23%)	2251 (28.35%)	149 (1.88%)	
350/µL-499/µL	1240 (15.62%)	1204 (15.17%)	36 (0.45%)	
≥ 500/µL	994 (12.52%)	976 (12.29%)	18 (0.23%)	
Peak CD4				< 0.010
< 100/µL	100 (1.26%)	37 (0.47%)	63 (0.79%)	
100/µL-199/µL	227 (2.86%)	134 (1.69%)	93 (1.17%)	
200/µL-349/µL	720 (9.07%)	593 (7.47%)	127 (1.60%)	
350/µL-499/µL	987 (12.43%)	901 (11.35%)	86 (1.08%)	
≥ 500/µL	5905 (74.38%)	5700 (71.80%)	205 (2.58%)	
Recent CD4				< 0.010
< 100/µL	125 (1.57%)	49 (0.62%)	76 (0.96%)	
100/µL-199/µL	323 (4.07%)	201 (2.53%)	122 (1.54%)	
200/µL-349/µL	961 (12.10%)	805 (10.14%)	156 (1.96%)	
350/µL-499/µL	1377 (17.34%)	1275 (16.06%)	102 (1.28%)	
≥ 500/µL	5153 (64.91%)	5035 (63.42%)	118 (1.49%)	

### Analysis of risk factors for mortality in PWH

In univariate Cox regression analysis, only peak CD4 was significantly associated with death and had a linear relationship with baseline CD4, peak CD4, and most recent CD4. Peak CD4 was then included in the multivariate survival analysis using the Cox regression model with other variables. After excluding confounders, three variables remained significantly associated with death, including male sex (hazard ratio (HR) = 1.268 [1.032, 1.559]; *P* = 0.024), time from HIV/AIDS confirmation to ART initiation ≥ 6 months (HR = 1.962 [1.631, 2.360]; *P* < 0.001), and peak CD4 count: CD4 < 100 cells/µL (HR = 16.093 [12.041, 21.508]; *P* < 0.001), 100 cells/µL ≤ x < 200 cells/µL (HR = 7.904 [6.148, 10.160]; *P* < 0.001), 200 cells/µL ≤ x < 350 cells/µL (HR = 3.166 [2.519, 3.980); *P* < 0.001), 350 cells/µL ≤ x < 500 cells/µL (HR = 1.668 [1.291, 2.155]; *P* < 0.001). The differences between the groups are shown in Table 3.

**Table 3:** Analysis of risk factors for death

	One-way analysis HR (95% CI)	*P*-value	Multifactorial analysis HR (95% CI)	*P*-value
Gender				
Female	1		1	
Male	1.676(1.371,2.048)	< 0.01	1.268 (1.032, 1.559)	0.024
Diagnosis to ART initiate interval				
< 6 months	1		1	
≥ 6 months	1.483 (1.239, 1.775)	< 0.01	1.962 (1.631, 2.360)	< 0.010
Marital Status				
Married /regular sexual partners	1			
Divorced/ widowed	0.739(0.568,0.961)	0.024		
Unmarried	2.333(1.878,2.898)	< 0.01		
Not available	1.800 (0.960, 3.377)	0.067		
Route of infection				
Heterosexual transmission	1			
Men Who Have Sex with Men	1.386 (0.911, 2.107)	0.127		
IDUs	1.370(0.901, 2.083)	0.140		
Mother-to-child transmission	5.044(4.133,6.156)	<0.01		
Not available	176.310(20.260,1534.342)	<0.01		
Peak CD4				
≥ 500/µL	1		1	< 0.010
350/µL-499/µL	1.634 (1.268, 2.107)	< 0.01	1.668 (1.291, 2.155)	< 0.010
200/µL-349/µL	2.979 (2.380, 3.729)	< 0.01	3.166 (2.519, 3.980)	< 0.010
100/µL-199/µL	7.518 (5.874, 9.621)	< 0.01	7.904 (6.148, 10.160)	< 0.010
< 100/µL	15.108 (11.386, 20.047)	< 0.01	16.093 (12.041, 21.508)	< 0.010
Baseline CD4				
≥ 500/µL	1			
350/µL-499/µL	1.202 (0.683, 2.117)	0.523		
200/µL-349/µL	1.724(1.057,2.814)	0.029		
100/µL-199/µL	2.208 (1.353, 3.604)	0.002		
< 100/µL	3.170 (1.959, 5.128)	< 0.01		
Recent CD4				
≥ 500/µL	1			
350/µL-499/µL	2.295 (1.759, 2.994)	< 0.01		
200/µL-349/µL	4.097 (3.216, 5.219)	< 0.01		
100/µL-199/µL	8.991 (6.959, 11.615)	< 0.01		
< 100/µL	20.942 (15.689, 27.954)	< 0.01		

### Correlation between peak CD4 count and mortality in PWH

**Fig. 2:**
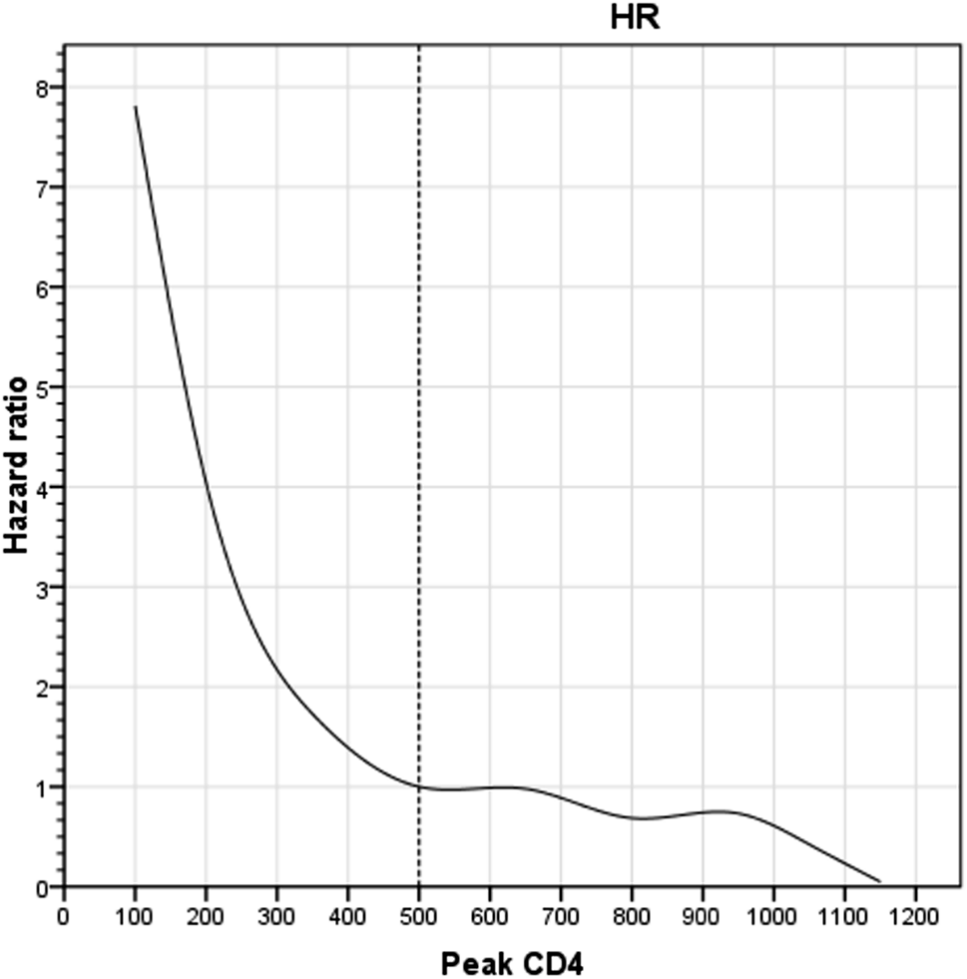
Fit curve of peak CD4 and hazard ratio

**Fig. 3:**
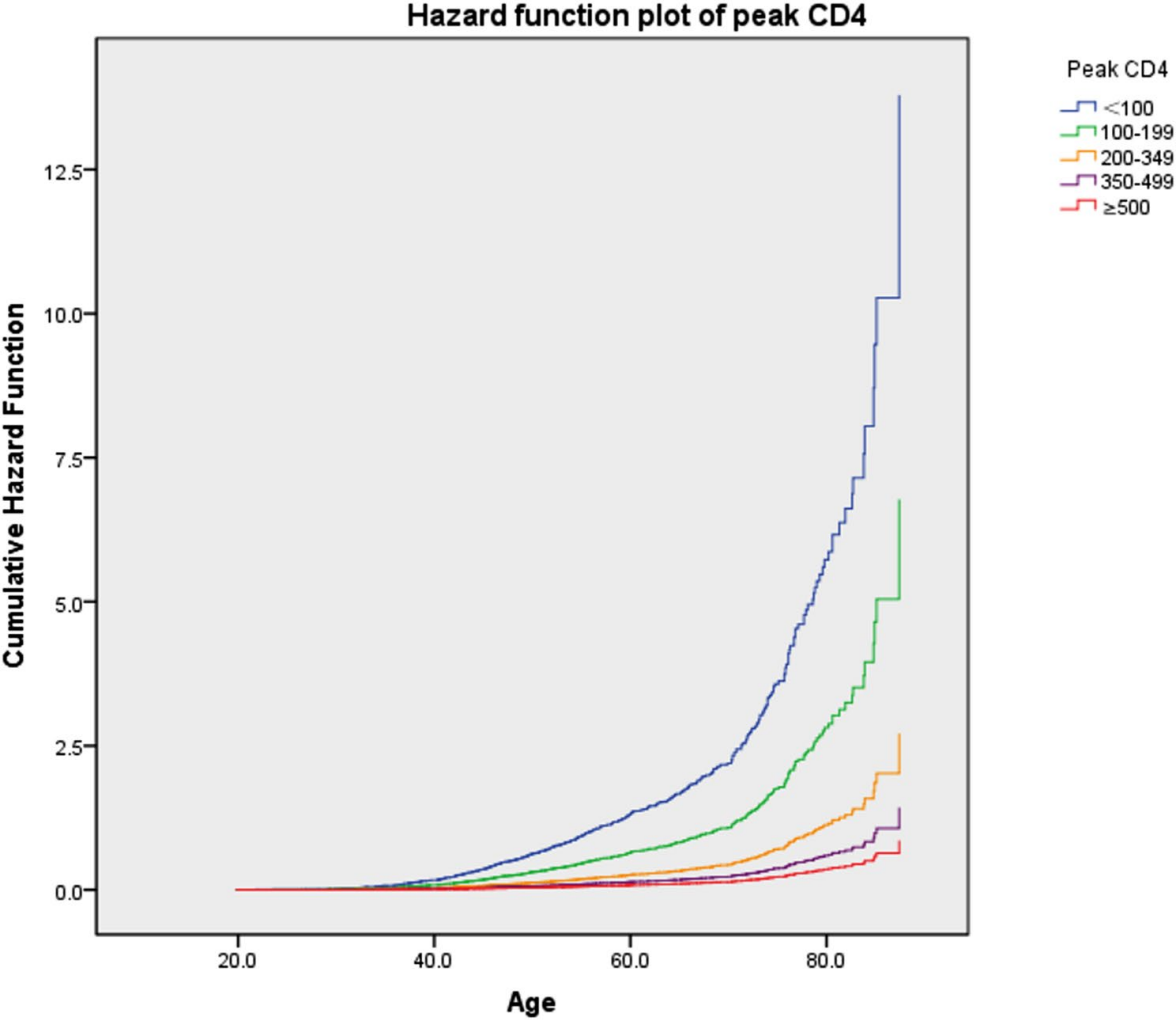
Cumulative hazard function plot of Peak CD4

It was shown that the risk of death in PWH increased with a peak CD4 cell count < 500 cells/µl, and the risk of death increased with a lower peak CD4 cell count (Fig. 2). When the peak CD4 cell count reached or exceeded 500 cells/µl, the HR slowly decreased from 1 and the peak CD4 count became a protective factor for mortality in PWH. Also, as can be clearly seen from the hazard function plot of peak CD4 (Fig. 3), when patients were the same age and had low CD4 counts, their cumulative risk of death was higher.The cumulative risk of death from HIV/AIDS did not show a statistically significant difference among patients between the ages of 20 and 40, but these differences gradually became more significant among patients over the age of 40. Details are shown in Fig. 3.

## Discussions

This retrospective cohort study investigated the association between peak CD4 cell count and mortality in PWH. By analyzing the data of 7939 PWH included in this study, we found that peak CD4 cell count was associated with the risk of death in PWH. Peak CD4 < 100cells/µL group (HR = 16.093 [12.041, 21.508]; *P* < 0.001), 100cells/µL ≤ x < 200cells/µL group (HR = 7.904 [6.148, 10.160]; *P* < 0.001), 200cells/µL ≤ x < 350cells/µL group (HR = 3.166 [2.519, 3.980]; *P* < 0.001), 350cells/µL ≤ x < 500cells/µL group (HR = 1.668 [1.291, 2.155]; *P* < 0.001).

When analyzing the demographic and clinical characteristics of the patients, our results are consistent with the findings of Trickey A [16] and Ingle SM [17] et al. that sex, marital status, and age had an impact on patient mortality. However, the results of a Cox regression analysis of transmission routes showed that differences in transmission routes did not increase the risk of death, which differs from the findings of Zhang G [18] et al. that IDUs with HIV had higher mortality rates. Chi-squared analysis of patients’ laboratory data showled that baseline CD4, peak CD4, or most recent CD4 cell count affected the survival status of PWH, i.e., low CD4 cell count had an impact on patient’ survival at any stage. The result of the study on baseline CD4 cell count is consistent with the studies by Wada [19] and Masiira B et al [20], who found that baseline CD4 cell count was associated with higher risk of mortality in a prospective cohort study. Meanwhile, in the analysis of 37,496 PWH with follow-up between 1996 and 2001, May [21] et al. after five years of ART, baseline CD4 counts become poor prognostic indicators.We hypothesized that the difference in outcome might be due to (1) the fact that the patients in this study were Chinese; (2) unlike the study by May et al, our study was designed as a retrospective study; (3) the adjusted variables in this study were inconsistent with the above-mentioned studies where they adjusted for sex, age, transmission risk, period of ART initiation, while we adjusted for the time from HIV diagnosis to ART initiation. The result of the most recent CD4 study is consistent with that of Young J [22] et al, who showed that higher CD4 cells (≥ 500 cells/µl) reduced mortality in PWH. In the Cox regression survival analysis of time from diagnosis to ART, we found that ART initiated < 6 months after HIV infection contributed to improved survival among patients. Several studies [23, 24] have shown that most PWH who do not receive ART typically die within two years, and our findings are consistent with World Health Organization guidelines. The analysis of peak CD4 in this study showed that a lower CD4 cell count was associated with an increased risk of death in patients with a peak CD4 cell count of less than 500 cells/µl. The risk of death for patients with a peak CD4 cell count < 100 cells/µL was 16 times higher than for patients with a peak CD4 cell count ≥ 500 cells/µL.

Early and rapid ART initiation strategy based on CD4 count has been reported to contribute to the prevention of HIV transmission and reduce the risk of several clinical outcomes [25, 26]. In PWH, CD4 cell count is one of the most important laboratory measures of immune function; when CD4 cell count drops below a certain level, PWH are at higher risk of immunodeficiency and are more susceptible to infections that can lead to advanced HIV disease (AHD) or death [27]. One study found that immediate initiation of ART in patients with a baseline CD4 cell count ≥ 500 cells/µL can lead to a significant reduction in AIDS-related mortality [28]. Our results underscore the importance of a CD4 cell count of ≥ 500 cells/µL for the survival of PWH at different stages. Detection of peak CD4 cell count may help clinicians to identify patients with poor immune reconstitution or treatment failure in a timely manner. Physicians should pay close attention to fluctuations in CD4 cell counts during ART, especially in patients with peak CD4 cell count that remains below 500 cells/µl (especially those with a peak CD4 cell count of less than 200cells/µL) or those with a CD4 cell count below baseline levels, in order to promptly monitor adherence to ART or the risk of virologic failure and to change their antiviral regimen if necessary. The findings of this study should be widely promoted in ART clinics that do not have access to HIV viral load testing, such as those in the resource-poor areas of Yunnan Province, together with advocacy for early diagnosis and treatment of HIV to protect PWH from severe immune dysfunction. While our study highlights the prognostic value of CD4 cell counts, it is critical to recognize that sustained viral suppression is the primary indicator of ART success. The relationship between CD4 count recovery and viral load reduction is complex and multifaceted. Although a higher CD4 count is indicative of immune recovery, the absence of detectable viral RNA in plasma is a direct marker of the efficacy of ART in controlling HIV replication. We suggest that future research and clinical monitoring strategies should prioritize viral load measurements while also considering CD4 count trends as part of a holistic approach to patient care.

The present study had several important advantages. First, the study had a large sample size (*n* = 7939) and a long follow-up period (the longest duration of sustained ART was 19 years). In addition, we examined the association between baseline CD4 cell count and recent survival status in PWH. Meanwhile, considering that the dynamic changes in CD4 cell count during follow-up may affect the survival status of PWH, we focused on exploring the relationship between peak CD4 cell count and survival status of PWH, which filled the current gap between peak CD4 and the risk of death from HIV. Finally, our study has some unavoidable limitations. Other risk factors (e.g., interruption of antiretroviral therapy, medication adherence, HIV viral load, CD4/CD8 ratio, etc.) that may affect mortality in HIV/AIDS were not included in this analysis because more detailed data were not available. In addition, our study population was drawn from a single treatment center, which may not be representative of all PWH, potentially limiting the generalizability of our results to other populations or settings with different socioeconomic backgrounds or health care systems.

## Conclusion

In conclusion, this observational study has higlighted the importance of peak CD4 cell count as a key indicator of immune recovery potential and survival outcomes in PWH on antiretroviral therapy. Our analysis showed that higher peak CD4 cell counts are associated with improved survival, suggesting that early diagnosis and timely initiation of ART aimed at achieving and maintaining higher CD4 count are critical to improving the prognosis of PWH. While recognizing the central role of sustained viral suppression as the primary marker of ART efficacy, our findings support the continued importance of monitoring CD4 cell counts as part of a comprehensive approach to patient care. These findings contribute to the growing body of evidence supporting the integration of both virologic and immunologic markers in the management and evaluation of ART outcomes. Future research should aim to further delineate the interplay between CD4 count dynamics, viral suppression and clinical endpoints to optimize treatment strategies for PWH.

## Data Availability

The datasets used and/or analyzed during the current study available from the corresponding author on reasonable request.
